# Minimal detectable change in inertial measurement unit-based trunk acceleration indices during gait in inpatients with subacute stroke

**DOI:** 10.1038/s41598-023-46725-5

**Published:** 2023-11-07

**Authors:** Tatsuya Igarashi, Yuta Tani, Ren Takeda, Tomoyuki Asakura

**Affiliations:** 1 Physical Therapy Division, Department of Rehabilitation, Numata Neurosurgery and Cardiovascular Hospital, Numata, Gunma Japan; 2 Department of Basic Rehabilitation, School of Health Sciences, Gunma University, Maebashi, Gunma Japan

**Keywords:** Health care, Medical research

## Abstract

Gait analysis using inertial measurement units (IMU) provides a multifaceted assessment of gait characteristics, but minimal detectable changes (MDC), the true change beyond measurement error, during gait in patients hospitalized with subacute stroke has not been clarified. This study aimed to determine the MDC in IMU-based trunk acceleration indices during gait in patients hospitalized with subacute stroke. Nineteen patients with subacute stroke (mean ± SD, 75.4 ± 10.9 years; 13 males) who could understand instructions, had a pre-morbid modified Rankin Scale < 3 and could walk straight for 16 m under supervision were included. As trunk acceleration indices, Stride regularity, harmonic ratio (HR), and normalized root mean square (RMS) during gait were calculated on three axes: mediolateral (ML), vertical (VT), and anterior–posterior (AP). MDC was calculated from two measurements taken on the same day according to the following formula: MDC = standard error of measurement × 1.96 × 2. The MDCs for each trunk acceleration index were, in order of ML, VT, and AP: 0.175, 0.179, and 0.149 for stride regularity; 0.666, 0.741, and 0.864 for HR; 4.511, 2.288, and 2.680 for normalized RMS. This finding helps determine the effectiveness of rehabilitation interventions in the gait assessment of patients with stroke.

## Introduction

One of the major disabilities after a stroke is a decreased ability to walk. It was reported that 82% of patients had not fully recovered the ability to walk at least 3 months after stroke^1^. Walking in patients with stroke is a critical ability related to the individual's quality of life^2,3^ and activities of daily living^4^. The temporal regularity of the gait cycle and the smoothness and efficiency of the center of gravity shifts are often impaired in walking after stroke, and these are closely related to balance function to prevent falls^5^. Decreased ability to walk after stroke results from the combined effects of motor paralysis, sensory dysfunction, and loss of balance control systems. Therefore, much of the rehabilitation of patients with stroke focuses on improving their ability to walk^6,7^.

Assessment of walking ability in the rehabilitation setting often involves visual observations and question-based clinical rating scales that rely on the expertise of the examiner. However, it has been noted that visual observational assessment of gait may not be sufficiently reliable^8^. Therefore, to accurately assess the gait of patients with stroke, it is advisable to use methods that can measure temporal and spatial parameters with high sensitivity, such as motion capture technology and inertial measurement devices. Gait analysis using an inertial measurement unit (IMU) can quantify gait characteristics such as smoothness of gait and regularity of steps derived from rhythmic motion during walking, which is difficult to evaluate with conventional gait analyzers and can evaluate gait characteristics from multiple perspectives. IMU is a highly feasible measurement method in clinical practice because of its portability and short measurement time^9^, reducing the patient’s burden. Furthermore, indicators obtained by walking with the IMU attached to the trunk (from now on collectively referred to as trunk acceleration indices) have been reported as associated with balance ability^5^ and trunk function^10^ after stroke, indicating their usefulness in gait assessment.

For the clinician to understand the clinical changes in a patient, the results measured by a tool must recognize minimal changes that are considered to be beyond simple measurement errors^11^. This minimum change is referred to as the minimal detectable change (MDC)^12^ or the smallest detectable change (SDC)^13,14^. Identifying the measures’ MDCs allows discrimination between measurement error and true change, which is a valuable insight in determining the effectiveness of rehabilitation interventions. MDC of trunk acceleration indices has been reported in healthy people^15^ and patients with chronic stroke^16,17^. However, the MDC of trunk acceleration indices during walking in inpatients with subacute stroke is yet to be clarified. Since the degree of ambulation due to recovery or compensation after stroke depends on the disease stage, clarifying the MDC of gait indices in early onset cases is a useful insight for clinicians to understand the clinical changes in their patients. This study clarifies the MDCs of trunk acceleration indices during walking in patients hospitalized with subacute stroke.

## Materials and methods

### Study design and participants

This was a single-center descriptive observational study. Data were collected from patients with stroke who were admitted consecutively to the general wards of Numata Neurosurgery and Cardiovascular Hospital between July 2022 and March 2023. This study was conducted under the approval of the Ethics Committees at Numata Neurosurgery and Cardiovascular Hospital, Japan (approval no.00168). Per the Declaration of Helsinki, all patients gave written informed consent before participation in this study. The authors had access to personally identifiable participant information during or after data collection.

This study included the following patients: (1) hospitalized with cerebral infarction or cerebral hemorrhage (first or recurrence), (2) with motor paralysis of the lower extremities, (3) able to walk 16 m without a walking aid, and (4) aged 20 years or older. This study excluded the following patients: (1) those unable to walk independently before the disease (pre-morbid modified Rankin Scale score (mRS) ≥ 3); (2) those who had difficulty communicating due to aphasia or cognitive decline. Since this study’s MDC is calculated from the Intraclass Correlation Coefficient (ICC), which is a reliability coefficient, the sample size was calculated based on the report by Walter et al.^18^. Referring to previous studies that reported MDCs for trunk acceleration indices^15^, the minimum acceptable ICC (p0) was set at 0.4 and the expected ICC (p1) was set at 0.8, and the required sample size, calculated by contingency table^18^, was 16 patients. The final sample size was 18 patients, considering a possible 10% attrition.

Demographic characteristics, including age, gender, body mass index, time since stroke, type of stroke (cerebral infarction or cerebral hemorrhage), mini-mental state examination, and pre-morbid mRS, were collected from medical records. Table 1 shows the participants’ demographic characteristics. There were 19 patients, most of whom were male and diagnosed with cerebral infarction. The mean MMSE score was 26.1 points, and patients had good cognitive function. FMA (lower limb) averaged 26.6 points, and most had mild motor paralysis in the lower limbs. Mini-BESTest averaged 19.6 points and had mild to moderate balance disorders.

**Table 1:** Demographics of participants.

Age (years), mean (SD)	75.4 (10.9)
Gender (male), n (%)	13.0 (68.4)
Body mass index (kg/m^2^), mean (SD)	22.2 (3.5)
Type of stroke (cerebral infarction), n (%)	17.0 (89.5)
Time since stroke (days), mean (SD)	23.7 (17.4)
Affected side (Rt), n (%)	13.0 (68.4)
MMSE, mean (SD)	26.1 (2.9)
Pre-morbid mRS (0/1/2), n	8/2/9
FAC (0/1/2/3/4/5), n	0/0/4/2/7/6
FMA (lower limb), mean (SD)	26.6 (2.9)
Mini-BESTest, mean (SD)	19.6 (6.0)
FIM, mean (SD)	114.9 (10.8)
CWS (m/s), mean (SD)	
Test	0.90 (0.27)
Retest	0.95 (0.24)

SD, standard deviation; MMSE, mini-mental state examination; mRS, modified Ranking Scale; FAC, functional ambulation categories; FMA, fugl meyer assessment; Mini-BESTest, mini-balance evaluation systems test; FIM, functional independence measure; CWS, comfortable walking speed.

### Data collection

All measurements were taken on the same day within a week before discharge. Trunk acceleration indices were measured as the primary outcome, and demographics and clinical characteristics were measured as secondary outcomes.

The trunk acceleration indices were measured by walking at a comfortable speed on a 10 m walking path with 3 m auxiliary paths at each end, for a total of 16 m straight path^19^. This was measured twice within 30 min using the same measurement method. The measurement was performed when the patient wore flat-soled shoes appropriate for their size. The trunk acceleration indices were measured using a capacitive 8-channel compact wireless motion recorder MVP-RF8-HC-2000 (Microstone Corporation, Nagano, Japan). The external dimensions of the device were 45 × 45 × 18 mm, and it weighed approximately 60 g. The sampling frequency was 200 Hz, and gravitational acceleration correction was performed. The band was attached at the height of the L3 spinous process to prevent the device from moving^20^. Acceleration signals were analyzed using dedicated software MVP-RF8-S (ver 1.7.7.0) (MicroStone Corporation, Nagano, Japan). Acceleration signals were transmitted in real-time via Bluetooth to a notebook PC equipped with dedicated software, digitally converted, and recorded as Excel data. The accelerometric variables were managed using Microsoft Excel for Mac version 16.31 (Microsoft Corp., Redmond, WA, USA). The laptop’s position was carefully considered since the maximum communication distance with the device was 30 m. Trunk acceleration indices were measured by a physical therapist who fully understood the measurement method. One physical therapist operated the laptop and visually checked for any abnormalities in the operation of the equipment during the measurement, while the other accompanied the patient to take measurements.

The numerical analysis software MATLAB (MathWorks Inc., Natick, MA, USA) was used to analyze the accelerometer variables. Each acceleration time series was filtered with a second-order Butterworth bandpass filter with a cutoff frequency of 0.1 to 20 Hz, and the processed, transformed values were used for analysis. Initial grounding was identified by characteristic and sharp peaks from the acceleration waveforms on the gait cycle's anterior–posterior (AP) axis^21^, and accelerometer variables were extracted for the central five gait cycles to analyze the steady-state gait data. The following trunk accelerometry variables were calculated; (1) stride regularity, an indicator to determine the regularity of stride^20^; (2) harmonic ratio (HR), an index of smoothness and stability of trunk movement during gait^22^; (3) normalized root mean square (RMS), an index of trunk sway^22^. These were obtained for the mediolateral (ML), vertical (VT), and AP axes.

Stride regularity was calculated by autocorrelating overlapping portions of the time series of acceleration values with duplicates of the same time series phase-shifted by the mean stride time^20^. The stride is the time from the ground contact of one foot to the ground contact of the next ipsilateral foot, and ground contact is determined by the characteristic sharp peak of the acceleration waveform on the AP axis^21^. Stride regularity values are distributed in a range from − 1 to 1, with closer to − 1 meaning less regularity and closer to 1 meaning more regularity. HR is an index calculated using the first 10 even harmonics (EH) and odd harmonics (OH) after the fast Fourier transform, respectively, assuming that the unit of measurement for continuous walking is stride (2 steps)^22^. VT and AP acceleration components consist of one peak per step, and ML acceleration components consist of one peak per two steps. Therefore, the HRs for VT and AP were calculated by dividing the sum of the acceleration values for EH by the sum of the acceleration values for OH, and the HR for ML was calculated by dividing the sum of the acceleration values for OH by the sum of the acceleration values for EH. RMS is an index that indicates the amplitude of acceleration, and it is interpreted that the larger the value, the larger the sway. It has been reported that the RMS of trunk acceleration during walking is affected by walking speed^22^. Therefore, in this study, we used the normalized RMS calculated by dividing by the square of the walking speed.

Clinical characteristics collected were Functional ambulation categories, a measure of walking independence^23^, Fugl-Meyer assessment-lower extremity as a measure of the severity of motor paralysis^24^, Mini-Balance Evaluation Systems Test as a measure of balance ability^25^, and functional independence measure^26^ as a measure of activities of daily living ability. In addition, a comfortable walking speed was calculated during the acceleration index measurement^19^.

### Data analysis

All statistical analyses were conducted using IBM SPSS Statistics, version 27 (IBM Corp., Armonk, N.Y., USA). Descriptive statistics were reported as mean ± standard deviation (SD) or frequency for patient demographic and clinical characteristics. The mean ± SD of the two measurements (test and retest) and the test–retest difference were calculated for the trunk acceleration indexes.

Next, the measurements’ reliability was first verified to clarify the MDC. The intra-rater reliability and its 95% confidence interval (95% CI) of the one-way variate model with ICC (1:1) were obtained for the test–retest measurements^27^. The criteria of Landis et al.^28^ were used to interpret ICCs; 0.81–1.00 was interpreted as almost perfect, 0.61–0.80 as substantial, 0.41–0.60 as moderate, 0.21–0.40 as fair, and 0.00–0.20 as slight. The standard error of measurement (SEM) was determined as SD × √(1 − ICC)^29^ to verify the measurements’ variability. Using these values, the final MDC was calculated using the following equation: MDC = SEM × 1.96 × 2^29^.

## Results

The descriptive statistics for each trunk acceleration indices are shown in Table 2. Of the three directions, the largest test–retest differences were in the ML direction for stride regularity, HR, and normalized RMS.

**Table 2:** Descriptive statistics for each trunk acceleration index.

	Test		Retest		Test–retest difference	
	Mean	SD	Mean	SD	Mean	SD
Stride regularity						
ML	0.437	0.155	0.470	0.126	− 0.033	0.091
VT	0.496	0.128	0.524	0.102	− 0.028	0.100
AP	0.508	0.112	0.513	0.112	− 0.005	0.084
HR						
ML	1.657	0.531	1.436	0.401	0.221	0.302
VT	1.781	0.525	1.674	0.453	0.107	0.415
AP	1.810	0.657	1.647	0.586	0.163	0.461
Normalized RMS						
ML	3.208	3.915	2.250	1.763	0.959	2.402
VT	4.132	3.354	3.744	2.997	0.389	1.154
AP	3.197	2.765	2.718	1.633	0.479	1.397

SD, standard deviation; ML, mediolateral; VT, vertical; AP, anteroposterior; HR, harmonic ratio; RMS, root mean square.

The results of the ICC and SEM for each trunk acceleration index are shown in Table 3. The ICC for each trunk acceleration index ranged from substantial to almost perfect for all indices.

**Table 3:** Results of intraclass correlation coefficient and standard error of measurement for each trunk acceleration index.

	ICC	95%CI		SEM
		Lower	Higher	
Stride regularity				
ML	0.775	0.512	0.906	0.063
VT	0.619	0.251	0.833	0.064
AP	0.734	0.439	0.888	0.054
HR				
ML	0.709	0.395	0.876	0.240
VT	0.637	0.279	0.842	0.267
AP	0.709	0.396	0.876	0.312
Normalized RMS				
ML	0.659	0.314	0.852	1.628
VT	0.930	0.832	0.972	0.826
AP	0.800	0.558	0.917	0.967

ICC, intraclass correlation coefficient; SEM, standard error of measurement; 95%CI, 95% confidence Interval; ML, mediolateral; VT, vertical; AP, anteroposterior; HR, harmonic ratio; RMS, root mean square.

Table 4 shows the MDC results for each trunk acceleration index. The mean MDC for each trunk acceleration index converged to 0.149–0.179 for stride regularity, 0.666–0.864 for HR, and 2.288–4.511 for normalized RMS.

**Table 4:** Results of minimal detectable change for each trunk acceleration index.

	MDC
Stride regularity	
ML	0.175
VT	0.179
AP	0.149
HR	
ML	0.666
VT	0.741
AP	0.864
Normalized RMS	
ML	4.511
VT	2.288
AP	2.680

MDC, minimal detectable change; ML, mediolateral; VT, vertical; AP, anteroposterior; HR, harmonic ratio; RMS, root mean square.

## Discussion

The trunk acceleration indices measured in this study during walking had moderate to almost perfect reliability. To our knowledge, this is the first study to reveal the MDC of trunk acceleration indices during gait in inpatients with subacute stroke. The results of this study may help clinicians determine the effectiveness of physical therapy interventions to improve walking ability in patients with subacute stroke.

MDC in normalized RMS was higher in ML than in VT and AP. Normalized RMS is widely used to measure trunk sway in the gait assessment of patients with stroke^5,16,30–32^. In addition, the trunk sway obtained by RMS after stroke is significantly different from that of healthy people, allowing for specific gait characteristics after stroke^31,32^. As in the previous study^32^, trunk sway in the lateral direction had the largest deviation compared to the other directions in this study. It has been reported that the reduced lateral stability of the hip joint that occurs in stroke patients affects the trunk's large lateral sway during gait and inadequate weight transfer during stance^33^. Lateral weight transfer capacity, related to hip abductor strength and trunk function, has been associated with gait speed in patients with subacute stroke^33^. The results also indicate that acceleration analysis helps characterize gait posture analysis because it can evaluate gait sway after stroke in the ML direction and any direction with moderate to almost perfect reliability.

HR calculated from trunk acceleration is a useful index that summarizes whole-body motion, unlike other stride regularity and normalized RMS^34^. HR has been reported to be associated with balance ability^5^ and has predictive validity for gait speed in patients with subacute stroke^35^. This study reveals MDC of HR in patients with subacute stroke, in addition to the previously reported patients with convalescent stroke^17^.

MDC values for stride regularity were generally similar to those reported for patients with chronic stroke^16^. Gait is a highly automated movement that is repeated at regular intervals^36^. However, after a stroke, motor paralysis, sensory impairment, and loss of balance may result in an asymmetric, energy-costly gait^37^. Therefore, a measure of gait regularity is an essential indicator of whether patients with stroke are acquiring efficient gait movements. The MDC value of stride regularity revealed in this study is a valuable index for determining the effectiveness of physical therapy interventions to obtain a regular gait cycle.

This study has several limitations. First, because the study included patients who could walk in a straight line under supervision or independently, many patients with mild motor paralysis and balance disorders were included. Second, only a sample from a single center was included in the study. In the future, having a sample of multiple centers would be desirable. Third, MDC values were only examined using one representative calculation method^29^, which should be kept in mind when interpreting the results. It would be desirable to clarify the MDC for each patient group in the future, considering patient characteristics such as function and age, including the severity of motor paralysis.

This study revealed MDC of trunk acceleration indices during walking in patients with subacute stroke. The results of this study may help clinicians determine the effectiveness of interventions to improve walking ability in patients with subacute stroke. For example, assume a situation where a patient with stroke is given gait rehabilitation and stride regularity, HR, and normalized RMS are measured as indicators of effectiveness. Referring to the MDCs obtained in this study will help determine whether the changes in gait resulting from rehabilitation are simply measurement errors or true changes. By identifying the meaning of change, it is possible to determine whether gait rehabilitation is an appropriate intervention for patients with stroke or whether intervention strategies need to be modified. This finding could help implement gait rehabilitation for stroke patients more efficiently.

## Data Availability

The data that support this study are available from the corresponding author upon reasonable request.
